# Sex‐based differences in shot put, javelin throw, and long jump in 8‐and‐under and 9–10‐year‐old athletes

**DOI:** 10.1002/ejsc.12241

**Published:** 2024-12-14

**Authors:** Gregory A. Brown, Brandon S. Shaw, Ina Shaw

**Affiliations:** ^1^ Physical Activity and Wellness Laboratory Department of Kinesiology and Sports Science University of Nebraska at Kearney Kearney Nebraska USA; ^2^ School of Sport Rehabilitation and Exercise Sciences University of Essex Colchester UK

**Keywords:** athletics, children, female, gender, male

## Abstract

There is an ongoing debate regarding the necessity for sex‐segregated sports particularly in youth. However, there has been minimal evaluation of prepubertal sex‐based differences in the events of shot put, javelin throw, and long jump. Therefore, the top eight performances from the USA Track and Field National Youth Outdoor Championships and National Junior Olympic Championships during the years 2016–2023 for shot put, javelin throw, and long jump in the 8‐and‐under and 9–10‐year‐old age groups were analyzed for sex‐based differences. The 8‐and‐under males threw the shot put farther (*P* < 0.0001 and Hedges’ *g* = 0.922) than females by 19.3% and the 9–10‐year‐old males threw the shot put farther (*P* = 0.016 and Hedges’ *g* = 0.332) than females by 6.5%. The 8‐and‐under males threw the javelin farther (*P* < 0.0001 and Hedges’ *g* = 1.269) than females by 32.6% and 9–10‐year‐old males threw the javelin farther (*P* < 0.0001 and Hedges’ *g* = 1.169) than females by 23.5%. The 8‐and‐under males long jumped farther (*P* = 0.010 and Hedges’ *g* = 0.359) than females by 4.7% and 9–10‐year‐old age males long jumped farther (*P* = 0.007 and Hedges’ *g* = 0.552) than females by 3.9%. The average between sex differences were larger than the within sex differences between the first through fourth place finishers in all but 9–10‐year‐old shot put. In all events, the greatest individual distance for a male exceeded that for a female. Therefore, the present data indicate that, in elite competition, males in the 8‐and‐under and 9–10‐year‐old age groups typically performed long jump and throw the shot put and javelin farther than females of the same age.

## Highlights

- Males in the 8‐and‐under age group outperformed females of the same ages by 19.3% in shot put, 32.6% in javelin throw, and 4.7% in long jump.
- Males in the 9–10‐year‐old age group outperformed females of the same ages by 6.5% in shot put, 23.5% in javelin throw, and 3.9% in long jump.
- The average differences between the first through fourth place finishers within males and within females were smaller than the sex‐based differences between males and females in all but 9–10‐year‐old shot put.

## 1 INTRODUCTION

In the consensus statement from the American College of Sports Medicine on the *Biological Basis of Sex Differences in Athletic Performance* (Hunter et al., 2023), sex is explained as the biological factors that are different between males and females, whereas gender refers to socially and culturally constructed roles and behaviors that relate to masculinity and femininity. The rationale and need for sex‐segregated sports have become a controversial topic in the past few years.

An argument against sex‐segregated sports is that sex segregation in sports propagates outdated gender stereotypes and is an unnecessary holdover of archaic social factors (Leong & Bartlett, 2019). An argument for sex‐segregated sports is that the biological differences between adult males and females give males inherent athletic advantages, thus sex‐segregated sports are necessary to protect the safety of female athletes and to provide for fair competition (Hilton & Lundberg, 2021; Hunter et al., 2023). The arguments for and against sex‐segregated sports become even more contentious when prepubertal children are involved because the focus on sports for these children is often recreation, physical activity, character building, and basic skill development (Wells & Arthur‐Banning, 2008). It is also unclear whether there are inherent differences between prepubertal males and females that influence competitive outcomes.

Physical fitness can be an important determinant of athletic success in adults and children (Armstrong & Van Mechelen, 2023; Malina et al., 2004; NSCA, 2017). However, it has been reported that there were no differences between 6‐ and 11‐year‐old males and females in tests of plank time, right knee extension force, modified pull‐ups, or grip strength (Ervin et al., 2014). In contrast, data from the Presidential Fitness Test, which was widely used in schools in the United States from the late 1950s until 2013, indicate that 8–10 years old males run faster in the 30 foot shuttle run, the mile run, and perform more curl‐ups and pull‐ups in one minute than females of the same ages (President’s Challenge Qualifying standards). There are also a number of studies that have found that, as young as 6 years old, males consistently and significantly outperform females of the same age on various tests of muscular strength, muscular power, aerobic fitness, and muscular endurance such as pushups, sit‐ups, bent arm hang, ball throw for distance, standing broad jump, sprint running, and distance running (Catley & Tomkinson, 2013; Tambalis et al., 2016; Thomas & French, 1985; Tomkinson et al., 2017, 2018; Vanhelst et al., 2020). When differences in physical fitness between prepubertal males and females are reported, the differences are much smaller than the differences after the onset of puberty (*ACSM's Guidelines for Exercise Testing and Prescription*, 2022; Malina et al., 2004; Armstrong & Van Mechelen, 2023), with corresponding smaller, if any, resulting differences in competitive performance.

It has been reported that there were not prepubertal sex‐based differences in competitive weightlifting performance (Huebner & Perperoglou, 2020; Mizuguchi et al., 2021). It has also been reported that the top 5 female swimmers under age 10 were faster than males and there were no differences between the 10th–50th ranked males and females under age 10 (Senefeld et al., 2019). In recent reviews on the topic of sex‐based difference in sports performance, the prepubertal sex‐based differences in sport performance have been described as “minimal” (Hunter et al., 2023) and “inconsequential or relatively small” (Bascharon et al., 2023). It has even been stated that it is only the increased testosterone secretion during puberty that causes a differentiation in athletic performance between males and females (Handelsman, 2017; Handelsman et al., 2018; Safer, 2022). However, there are other sources indicating that the differences in athletic performance between prepubertal males and females are statistically significant and may be large enough to preclude prepubertal females from earning victory in head to competition with males of the same age group.

Sources indicating that prepubertal males are faster and throw and jump farther than prepuberal females include records of all‐time best performances and scholarly papers. The overall youth records for all‐time best performances from the USA Track and Field (USATF) as of December 19, 2018 (USATF, 2018), from the USATF National Junior Olympic Track and Field Championships as of March 27, 2019 (USATF, 2019), the Amateur Athletic Union (AAU) as of November 22, 2023 (AAU, 2023), and School Sport Australia as of December 2016 (School Sport Australia, 2016) indicate that males outperform females in running, shot put, javelin throw, and long jump. Also, the 10‐and‐under records for all‐time best performances from the USA Swimming (as of November 22, 2023) indicate that males are 1.8% faster than females in 11 out of 12 individual short course events and 9 out of 11 individual long course events (USA Swimming, 2023). There are also two scholarly evaluations indicating prepubertal sex‐based differences in athletic performance. It has recently been reported that males in the 8‐and‐under and 9–10‐year‐old age groups in the USATF national level meets run faster than similarly aged females by 2.9%–6.7% in events of 100, 200, 400, 800, and 1500 m (Brown et al., 2024). Similarly, Atkinson et al. (Atkinson et al., 2024) have reported that the top 50 track running performances in 7–12‐year‐old males were ∼4.0% faster than same aged females in 100, 200, 400, and 800 m. These same authors also reported that the top 50 performances in 7–12‐year‐old males were ∼6.8% farther in long jump and ∼5.3% higher for high jump compared to same age females. Also showing prepubertal sex‐based differences in athletic performance, Handelsman (2017) states that prepubertal males run 3.0% faster and jump 5.8% farther than females but the author subsequently concluded that sex‐based differences in athletic performance do not arise until 12–13 years of age. Although male sex‐based differences in throwing are well known and have been reported to be present by 3 years of age (Emeljanovas et al., 2020; Gromeier et al., 2017; Liu & Zhao, 2022; Thomas & French, 1985), these evaluations have focused on field tests of distance, speed, or accuracy rather than competitive throwing events such as shot put and javelin throw.

Given the disagreement about whether there are differences in athletic performance before puberty, an evaluation of competitive throwing and jumping in prepubescent children is warranted. Therefore, the purpose of this project was to determine if there are sex‐based differences in shot put, javelin throw, and long jump in athletes under age 10.

## 2 METHODS

The eight best distances for males and females for the USATF National Youth Outdoor Championships and the USATF National Junior Olympic Championships during the years 2016–2023 for shot put, javelin throw, and long jump in the 8‐and‐under age group were downloaded in 1‐year brackets from the Athletic.net website between January 1 and August 12, 2023. All data were transcribed from the Athletic.net datasets into a spreadsheet (Excel 365, Microsoft Inc., Redmond, WA) by the same researcher (G.A.B.). All data in the spreadsheet were then cross checked for accuracy with Atletic.net data, and corrected where necessary, by two office associates who were not affiliated with this project. The USATF National Youth Outdoor Championships and the USATF National Junior Olympic Championships were not held in 2020 due to the COVID‐19 pandemic. The eight best distances were selected as these correspond to the finalist heat in running events. The same procedures were repeated for the 9–10‐year‐old age group. With three events with eight distances per event for two age groups and two sexes over seven years in two meets per year, there were potentially 1344 distances for data analysis. Due to there not being eight athletes with distances for each event each year, there were 1237 finishing distances included in the data analysis (Table 1). The 8‐and‐under and 9–10‐year‐old age groups represent the youngest age divisions used in USATF events. The USATF National Youth Outdoor Championships and USATF National Junior Olympic Championships were selected for comparison as these track meets should represent a sampling of elite youth athletes in shot put, javelin throw, and long jump from across the USA and the meets should be conducted using uniform policies regarding eligibility and measuring procedures. The 2 kg shot was used for both sexes for the 8‐and‐under age group, whereas the 6 pound shot was used for both sexes for the 9–10‐year‐old age group. The 300 g training javelin was used for both sexes and both age groups. All procedures for this project accessed public information and did not require ethical review in accordance with the Code of Federal Regulations, 45 CFR 46.102, and the Declaration of Helsinki.

**TABLE 1:** Events and number of distances analyzed in the 8‐and‐under and 9–10‐year‐old age groups from the USA Track and Field National Youth Outdoor Championships and the USA Track and Field National Junior Olympic Championships during the years 2016–2023 (These track meets were not held in 2020 due to the COVID‐19 pandemic).

Event	Age group		Males		Females
		n	Explanation for why n ≠ 112 (if necessary)	n	Explanation for why n ≠ 112 (if necessary)
Shot put	8‐and‐under	94	Only 4 participants in 2017 in YC and 1 scored DNS	90	1 participant scored DNS in 2016 in YC
			Only 7 participants in 2018 in YC and 1 scored DNS		Only 5 participants in 2017 in YC and 3 scored DNS
			Only 5 participants in 2019 in YC		Only 6 participants in 2018 in YC
			Only 6 participants in 2021 in YC and 1 scored DNS		Only 4 participants in 2019 in YC and 1 scored DNS
			Only 7 participants in 2022 in YC and 1 scored DNS		Only 3 participants in 2021 in YC
			Only 6 participants in 2023 in YC and 1 scored DNS		Only 6 participants in 2023 in YC and 1 scored DNS
	9–10‐year‐old	112		98	3 DNS in 2017 in YC
					1 participant scored DNS in 2018 in YC
					Only 5 participants in 2019 in YC
					Only 5 participants in 2021 in YC and 1 scored DNS
					Only 7 participants in 2023 in YC and 3 scored DNS
Javelin throw	8‐and‐under	100	Only 1 participant in 2017 in YC	97	Only 5 participants in 2017 in YC and 1 scored DNS
			Only 7 participants in 2019 in YC		Only 4 participants in 2018 in YC
			Only 7 participants in 20121 in YC and 2 scored DNS		Only 5 participants in 2019 in YC and 2 scored DNS
			1 scored DNS in 2023 in YC		Only 6 participants in 2021 in YC and 1 scored DNS
	9–10‐year‐old	110	2 scored DNS in 2019 in YC	105	Only 4 participants in 2019 IN YC and 1 scored DNS
					Only 7 participants in 2021 in YC and 2 scored DNS
Long jump	8‐and‐under	112		101	Only 6 participants in 2019 in YC and 3 scored DNS
					2 scored DNS in YC in 2022
					Only 5 participants in 2023 in YC and 1 scored DNS
	9–10‐year‐old	110	2 DNS in 2023 in YC	112	

*Note*: The distances were taken from the top eight performances.

DNS = did not start. YC = USA Track and Field National Youth Outdoor Championships.

*Calculations and Statistics*. Data are presented as means ± standard deviation. All distances are reported in meters. Data for males and females in each group were compared using a 2‐tailed 2‐sample unequal variance *t*‐test (SigmaStat 4.0, Systat Software, San Jose, CA). Further, 2‐tailed 2‐sample unequal variance *t*‐tests comparing the top 10 performances for each event and age group were also conducted to compare the best male and female distances. Effect size was calculated using Hedges’ g since the sample sizes in each sex were not always the same. Percent differences between the male and female performances in each event were calculated using the equation described by Millard–Stafford (Millard‐Stafford et al., 2018).

$$\frac{Male\;Distance-Female\;Distance}{Male\;Distance}X\;100$$

Percent differences between the first versus the second place, the second versus the third place, and the third versus the fourth place were calculated to compare between sex differences of the medal winning performances to within sex differences using the equation below, which was derived from the equation used by Millard‐Stafford et al. (2018).

$$\frac{{\;distance\;for\;place\;n-distance\;for\;place\;n}_{-1}}{{distance\;for\;place\;n}_{-1}}X\;100$$

## 3 RESULTS

**FIGURE 1:**
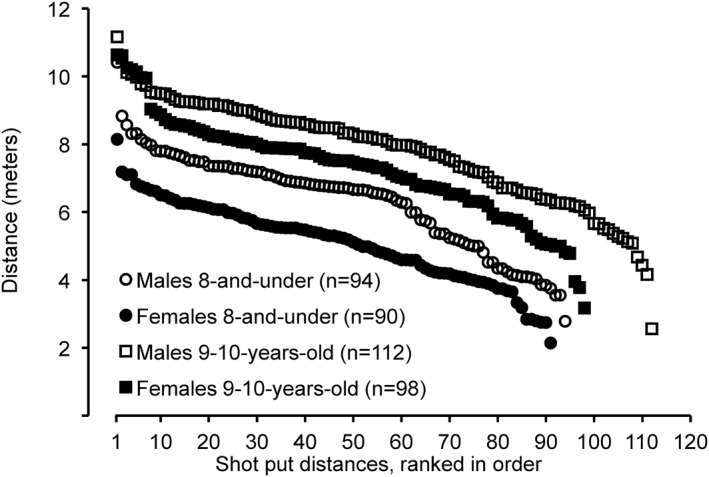
Shot put distances from longest to shortest for the top 8 performances in the 8‐and‐under and in the 9–10‐year‐old age groups from the USA Track and Field National Youth Outdoor Championships and the USA Track and Field National Junior Olympic Championships during the years 2016–2023*. *P* < 0.0001 for 8‐and‐under males versus females, effect size (Hedges’ g) = 0.922. *P* = 0.016 for 9–10‐year‐old males versus females, effect size (Hedges’ g) = 0.332. *These track meets were not held in 2020 due to the COVID‐19 pandemic.

*Shot put*. Shot put distances for 8‐and‐under males (6.33 ± 1.44 m) were 19.3% farther (*P* < 0.0001 and Hedges’ *g* = 0.922) than for 8‐and‐under females (5.11 ± 1.19 s). The range of shot put distances for 8‐and‐under males was 2.78–10.41 m (Figure 1), whereas the range for 8‐and‐under females was 2.14–8.14 m. The top 10 shot put distances for males in the 8‐and‐under age group (8.42 ± 0.77 m) were farther (*P* < 0.0001) than for females (6.95 ± 0.48 m). The longest shot put distance for a male was 21.8% farther than for a female. Five males in the 8‐and‐under age group had longer shot put distances than the longest distance for a female in the 8‐and‐under age group. The average percent difference between the first through fourth place shot put finishers for males and females (combined) in the 8‐and‐under age group was 8.8 ± 3.4%.

Shot put distances for 9–10‐year‐old males (7.77 ± 1.53 m) were 6.5% farther (*P* = 0.016 and Hedges’ *g* = 0.332) than for 9–10‐year‐old females (7.27 ± 1.48 m). The range of shot put distances for 9–10‐year‐old males was 2.56–11.16 m (Figure 1), whereas the range for 9–10‐year‐old females was 3.17–10.63 m. The top 10 shot put distances for males in the 9‐10‐year‐old age group (9.99 ± 0.77 m) were not different (*P* = 0.599) than for females (9.85 ± 0.66 m). The longest shot put distance for a male was 4.7% farther than for a female. One male in the 9–10‐year‐old age group had a longer shot put distance than longest distance for a female in the 9–10‐year‐old age group. The average percent difference between the first through fourth place shot put finishers for males and females (combined) in the 9–10‐year‐old age group was 8.3 ± 3.2%.

**FIGURE 2:**
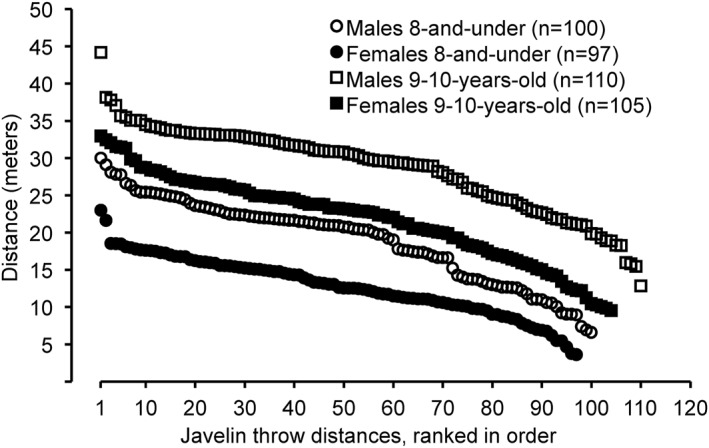
Javelin throw distances from longest to shortest for the top 8 performances in the 8‐and‐under and in the 9–10‐year‐old age groups from the USA Track and Field National Youth Outdoor Championships and the USA Track and Field National Junior Olympic Championships during the years 2016–2023*. *P* < 0.0001 for 8‐and‐under males versus females, effect size (Hedges’ g) = 1.269. *P* < 0.0001 for 9–10‐year‐old males versus females, effect size (Hedges’ g) = 1.169. *These track meets were not held in 2020 due to the COVID‐19 pandemic.

*Javelin throw*. Javelin throw distances for 8‐and‐under males (19.00 ± 5.64 m) were 32.6% farther (*P* < 0.0001 and Hedges’ *g* = 1.269) than for 8‐and‐under females (12.81 ± 3.94 m). The range of javelin throw distances for 8‐and‐under males was 6.60–29.99 m (Figure 2), whereas the range for 8‐and‐under females was 3.62–22.98 m. The top 10 javelin throw distances for males in the 8‐and‐under age group (26.75 ± 1.34 m) were farther (*P* < 0.0001) than for females (18.39 ± 1.20 m). The longest javelin throw distance for a male was 23.4% farther than for a female. Twenty‐four males in the 8‐and‐under age group had longer javelin throw distances than the longest distance for a female in the 8‐and‐under age group. The average percent difference between the first through fourth place javelin throw finishers for males and females (combined) in the 8‐and‐under age group was 13.2 ± 4.0%.

Javelin throw distances for 9–10‐year‐old males (28.57 ± 5.74 m) were 23.5% farther (*P* < 0.0001 and Hedges’ *g* = 1.169) than for 9–10‐year‐old females (27.87 ± 5.72 m). The range of javelin throw distances for 9–10‐year‐old males was 12.83–44.18 m (Figure 2), whereas the range for 9–10‐year‐old females was 9.52–32.93 m. The top 10 javelin throw distances for males in the 9–10‐year‐old age group (35.80 ± 1.37 m) were farther (*P* < 0.0001) than for females (30.42 ± 1.251 m). The longest javelin throw distance for a male was 25.5% farther than for a female. Twenty‐nine males in the 9–10‐year‐old age group had longer javelin throw distances than the longest distance for a female in the 9–10‐year‐old age group. The average percent difference between the first through fourth place javelin throw finishers for males and females (combined) in the 9–10‐year‐old age group was 8.8 ± 4.2%.

**FIGURE 3:**
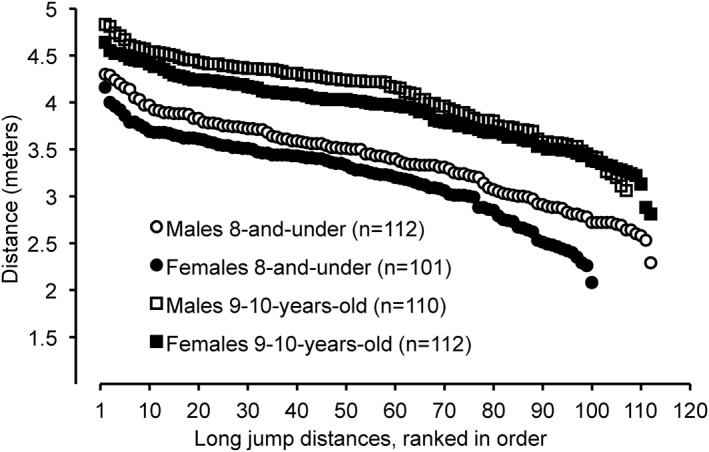
Long jump distances from longest to shortest for the top 8 performances in the 8‐and‐under and in the 9–10‐year‐old age groups from the USA Track and Field National Youth Outdoor Championships and the USA Track and Field National Junior Olympic Championships during the years 2016–2023*. *P* = 0.010 for 8‐and‐under males versus females, effect size (Hedges’ g) = 0.359. *P* = 0.007 for 9–10‐year‐old males versus females, effect size (Hedges’ g) = 0.552. *These track meets were not held in 2020 due to the COVID‐19 pandemic.

*Long jump*. Long jump distances for 8‐and‐under males (3.38 ± 0.45 m) were 4.7% farther (*P* = 0.010 and Hedges’ *g* = 0.359) than for 8‐and‐under females (3.22 ± 0.44 m). The range of long jump distances for 8‐and‐under males was 2.29–4.30 m (Figure 3), whereas the range for 8‐and‐under females was 2.08–4.16 m. The top 10 long jump distances for males in the 8‐and‐under age group (4.14 ± 0.12 m) were farther (*P* < 0.0001) than for females (3.87 ± 0.14 m). The longest long jump distance for a male was 3.3% farther than for a female. Four males in the 8‐and‐under age group had longer long jump distances than the longest distance for a female in the 8‐and‐under age group. The average percent difference between the first through fourth place long jump finishers for males and females (combined) in the 8‐and‐under age group was 4.2 ± 3.8%.

Long jump distances for 9–10‐year‐old males (4.05 ± 0.45 m) were 3.8% farther (*P* = 0.007 and Hedges’ *g* = 0.552) than for 9–10‐year‐old females (3.90 ± 0.38 m). The range of long jump distances for 9–10‐year‐old males was 2.54–4.83 m (Figure 3), whereas the range for 9–10‐year‐old females was 2.81–4.64 m. The top 10 long jump distances for males in the 9–10‐year‐old age group (4.67 ± 0.10 m) were farther (*P* < 0.0001) than for females (4.49 ± 0.07 m). The longest long jump distance for a male was 3.9% farther than for a female. Five males in the 9–10‐year‐old age group had longer long jump distances than the longest distance for a female in the 9–10‐year‐old age group. The average percent difference between the first through fourth place long jump finishers for males and females (combined) in the 9–10‐year‐old age group was 3.0 ± 1.3%.

## 4 DISCUSSION

In this evaluation of the top 8 distances in shot put, javelin throw, and long jump in the USATF National Youth Outdoor Championships and Junior Olympic National Championships from 2016 to 2023, males in the 8‐and‐under and 9–10‐year‐old age groups had farther average distances than females and the longest distances for males were farther than the longest distances for females. These sex‐based differences in throwing and jumping performance corresponded with large effects sizes for shot put in the 8‐and‐under age group and javelin throw in the in the 8‐and‐under and in the 9–10‐year‐old age groups and with a medium effect size for long jump in the 9–10‐year‐old age group, with the remaining effects sizes being between small and medium. Furthermore, the average differences between males and females for shot put, javelin throw, and long jump were larger than the within sex differences between the first through fourth place finishers except for the 9–10‐year‐old shot put. Also, there was no difference in shot put distance between the top 10 males and females in the 9–10‐year‐old age group but there were for the other events and age groups. Taken together, these data provide information on the presence and absence of sex‐based differences in sports performance that can be useful when deciding if sex‐segregated sports are necessary.

To the best of our knowledge, this is the first evaluation of sex‐based differences in competitive shot put performance in athletes 10 years old and younger. The current data indicate that the average distances for shot put are farther for males than females by 19.3% in the 8‐and‐under and 6.5% farther in the 9–10‐year‐old age groups. The current data also indicate that the longest distance for a male in the 8‐and‐under age group was 21.8% farther than for a female and the longest distance for a male in the 9–10‐year‐old age group was 4.7% farther than for a female. The difference in performance between the top 4 finalists for males and females combined was 8.8% for the 8‐and‐under age group and 8.3% for the 9–10‐year‐old age group. In the present data, there was no significant difference in shot put distance for the top 10 males and females in the 9–10‐year‐old age group but there was in the 8‐and‐under age group. In comparison, the records for all‐time best shot put performances in the 8‐and‐under age group from the USATF Junior Olympics as of March 27, 2019 (USATF, 2019), USATF as of December 19, 2018 (USATF, 2018), and the AAU Junior Olympics as of November 22, 2023 (AAU, 2023) indicate that distances for males in the 8‐and‐under age group were 12.5%, 13.6%, and 18.2% (respectively) farther than for females. The records for all‐time best shot put performances in the 9–10‐year‐old age group from these same sources (AAU, 2023; USATF, 2018; USATF, 2019) indicate that distances for males in the 9–10‐year‐old age group were 21.9%, 21.4%, and 18.9% (respectively) farther than for females. The record for all‐time best shot put performances in 10‐year‐old children from School Sport Australia indicate that the male record is 9.3% farther than the female record (School Sport Australia). The sex‐based differences in shot put distance for the 8‐and‐under age group in the present data are reasonably close to the sex‐based differences in records of all‐time best performances and in fitness tests that involve throwing. However, the sex‐based differences in shot put distance for the 9–10‐year‐old age group in the present data are lower than the sex‐based differences in records of all‐time best shot put performances. The longest shot put distance for a 9–10‐year‐old female in the present data includes a distance of 10.63 m, which exceeds the all‐time records from USATF (USATF, 2018), the USATF Junior National Olympics (USATF, 2019), and the AAU (AAU, 2023), which are 10.46 m, 10.53 m, and 10.36 m (respectively) and this distance occurred after the last update to the USATF record lists. In contrast, the longest distance for a 9–10‐year‐old male in the present data is 11.16 m, which is shorter than the all time records for these same sources (AAU, 2023; USATF, 2018; USATF, 2019), which indicate that the longest shot put distances for males in the 9–10‐year‐old age group were 13.40 m, 13.40 m, and 12.90 m (respectively). The records for all‐time best shot put performances in 10‐year‐old children from School Sport Australia indicates that the male record is 14.43 m and the female record is 13.21 m, but these were achieved with a 2 kg shot (School Sport Australia), whereas USA Track and Field uses a 6 pounds shot for the 9–10‐year‐old age group. The significant difference in shot put distance between the top 10 8‐and‐under males and females, but no difference in shot put distance between the top 10 9–10‐year‐old males and females is intriguing and may be caused by the earlier age of onset of puberty in females (Hunter et al., 2023). Unfortunately, since the present data only include the age group and sex of the participants, it is not possible to explain the causes for the presence or absence of sex‐based differences in performance.

To the best of our knowledge, this is also the first evaluation of sex‐based differences in competitive javelin throw performance in children 10 years of age and younger. The current data indicate that the average distances for javelin throw are farther for males than females by 32.6% in the 8‐and‐under and 23.5% farther in the 9–10‐year‐old age groups. The current data also indicate that the longest distance for a male in the 8‐and‐under age group was 23.4% farther than for a female and the longest distance for a male in the 9–10‐year‐old age group was 25.5% farther than for a female. The difference in performance between the top 4 finalists for males and females combined was 13.2% for the 8‐and‐under age group and 8.8% for the 9–10‐year‐old age group. In the present data, there was a significant difference in javelin throw distance for the top 10 males and females in the 9–10‐year‐old age group and in the 8‐and‐under age group. In comparison, the records for all‐time best javelin throw performances from the USATF Junior Olympics as of March 27, 2019 (USATF, 2019), USATF as of December 19, 2018 (USATF, 2018), and the AAU Junior Olympics as of November 22, 2023 (AAU, 2023) indicate that the distances for males in the 8‐and‐under age group were 30.5%, 27.8%, and 20.3% (respectively) farther than for females. The records for all‐time best javelin throw performances from these same sources (AAU, 2023; USATF, 2018; USATF, 2019) indicate that the distances for males in the 9–10‐year‐old age group were 17.2%, 15.9%, and 11.0% (respectively) farther than for females. The sex‐based differences in javelin throw distance in the present data are similar to the differences shown in records of all‐time best performance USATF and AAU (the records from School Sport Australia do not include javelin throw). The sex‐based differences in javelin throw in both the 8‐and‐under and 9–10‐year‐old age groups in the present data and records of all‐time best performance do not provide any insight into whether maturation, puberty, or factors other than sex and age group influence javelin performance.

Supporting the present findings of sex‐based difference in shot put and javelin throw performance in children 10 years old and younger, physical fitness testing and other evaluations of throwing performance indicate that males throw farther than females starting at even younger ages than in the present investigation. Greater throwing velocity, which undoubtedly translates into greater throwing distance, was observed by Thomas and French (1985) who indicate that throwing velocity in males exceeds that of females by 1.5 standard deviation units as young as 4 years old and increases to 3.5 standard deviation units by age 12. In fitness tests that involve throwing, Emeljanovas et al. (2020) observed that 6–8‐year‐old males throw a tennis ball 23.5% farther and have 14.8% better performance on the medicine ball push than females of the same ages, whereas 9–10‐year‐old males throw a tennis ball 25.8% farther and have 12.1% better performance on the medicine ball push than females of the same ages. Similarly, Catley et al. (Catley & Tomkinson, 2013) observed that the distances for 9–10‐year‐old males performing a basketball‐throwing test were 8.1% farther than females of the same age. Gromeier et al. (2017) indicate that, prior to puberty, male children exhibit more effective stepping and trunk movement while throwing. Even when young females are coached for better overhand throwing technique, their performance on tests of throwing distance and throwing velocity improve their performance still does not equalize to that of males (Lombardo & Deaner, 2018). It is interesting to note that Liu and Zhao (2022) indicate that the sex‐based differences in throwing performance have remained consistent for the past 40 years.

The 8‐and‐under males and females used throwing implements of the same size and mass, as did the 9–10‐year‐old males and females, allowing for an equal comparison between the sexes. The current adult male world record shot put distance is 23.56 m, whereas the adult female world record is 22.63 m (World Athletics, 2023). However, adult males use a 7.26 kg shot, whereas adult females use a 4 kg shot, which makes it difficult to compare adult males and females in this event other than to say that the male world record is 0.93 m (a difference of 3.9%) using an 81.5% heavier throwing implement. Similarly, the adult male world record for javelin throw is 98.48 m and the female world record is 72.28 m. The javelin used by adult females must weigh at least 600 g and be 2.2–2.3 m long, whereas the javelin used by adult males must weigh at least 800 g and be 2.6–2.7 m long. The difference in throwing implement size makes it difficult to directly compare javelin throw distances in adult males and females other than to say that the male world record javelin throw distance is 26.2 m farther than the female world record (a difference of 26.6%) even though males use an implement that is 200 g heavier and 0.4 m longer. Thus, although adult males perform better than adult females in shot put and javelin throw and the present data and data for all‐time best performances indicate that in children age 10 years old and younger males perform better than females of the same age, it is not possible to make a straightforward comparison of how the size of the sex‐based differences in shot put and javelin throw change from childhood through adulthood, which may be useful when making decisions regarding the necessity of sex‐segregated sports.

In the present data, the average long jump distances for 8‐and‐under males were 4.7% farther than females and were 3.8% farther for 9–10‐year‐old males than females. The current data also indicate that the longest long jump distance for a male in the 8‐and‐under age group was 3.3% farther than for a female and the longest distance for a male in the 9–10‐year‐old age group was 3.9% farther than for a female. The difference in performance between the top 4 finalists for males and females combined was 4.2% for the 8‐and‐under age group and 3.0% for the 9–10‐year‐old age group. In the present data, there was a significant difference in the long jump distance for the top 10 males and females in the 9–10‐year‐old age group and in the 8‐and‐under age group. Handelsman (2017) pooled together data on high jump, pole vault, long jump, triple jump, and standing long jump performance and reported a 5.8% prepubertal sex‐based difference in jumping. Atkinson et al. (2024) observed that males aged 7–12 years old long jumped farther than same aged females by ∼5.7%. The present sex‐based differences in the long jump performance are smaller than what was reported for all jumping events by Handelsman, but it is not possible to directly compare the present long jump data to the pooled jumping data analyzed by Handelsman. The present sex‐based differences in long jump are also smaller than those reported by Atkinson. The records for all‐time best long jump performances from the USATF Junior Olympics as of March 27, 2019 (USATF, 2019), USATF as of December 19, 2018 (USATF, 2018), and the AAU Junior Olympics as of November 22, 2023 (AAU, 2023) indicate that the distances for males in the 8‐and‐under age group were 16.4%, 10.5%, and 7.6% (respectively) farther than for females. The records for all‐time best long jump performances in the 9–10‐year‐old age group from these same sources (AAU, 2023; USATF, 2018; USATF, 2019) indicate that the distances for males were 6.0%, 5.9%, and 5.3% (respectively) farther than for females. Records of all‐time best performance for School Sport Australia (School Sport Australia) indicate that 10‐year‐old males jump 7.6% farther than females of the same age. Collectively, the present and previous data indicate that males aged 10‐and‐under performed long jump farther than same aged females, with the present data having a smaller difference between the sexes than other sources. In physical fitness tests of jumping, the sex‐based differences for the 8‐and‐under age group range from 8.3% to 9.1%, whereas for the 9–10‐year‐old age group, the differences range from 7.0% to 8.2% (Catley & Tomkinson, 2013; Emeljanovas et al., 2020; Tambalis et al., 2016; Tomkinson et al., 2018). It is unclear why the sex‐based differences in long jump in the present data are smaller than the sex‐based differences in records of all‐time best performances, previous research, and in fitness tests of jumping.

The present analysis of real world data are what has been described by Hunter (2024) as a “Top Down Approach,” which can provide insight into the age and sex‐based differences in athletic performance. The sex‐based differences in shot put, javelin throw, and long jump in children ages 10‐and‐under range from 3.9% in shot put for the 9–10‐year‐old group to 32.6% in javelin throw in the 8‐and‐under age group. Handelsman (2024) has stated that a sex‐based margin of victory in adults greater than one percent could be considered unfair when competing against “individual or identifiable groups of competitors”. In contrast, Pike (2023) has argued that the magnitude of advantage is irrelevant when one considers that sex represents a defined category with known male advantage. Although the present data indicate that there are sex‐based differences in competitive shot put, javelin throw, and long jump in children ages 10‐and‐under, the present data do not explain the underlying causes of these differences other than age group and sex. However, there a number of biological and social factors that may influence sports performance. Success in throwing and jumping events can be broadly broken down to the factors of muscle strength, power, speed, and technique (Caughey & Thomas, 2022; Hay, 1986; Mastalerz & Sadowski, 2022). Although it has been reported that there are no prepubertal sex‐based differences in muscle strength (Ervin et al., 2014), other sources indicate that prepubertal males have greater muscle strength than females of the same age (Catley & Tomkinson, 2013; Tambalis et al., 2016; Tomkinson et al., 2017, 2018; Vanhelst et al., 2020). Lean body mass is an important determinant of muscle strength and reports indicate that prepubertal males have ∼10% more lean body mass than females (McManus & Armstrong, 2011; Staiano & Katzmarzyk, 2012). McManus and Armstrong (McManus & Armstrong, 2011) also report that before puberty males have larger and more efficient hearts and lungs than females, which can facilitate better athletic performance. There are also differences in the width of the ischium and acetabular regions of the pelvis between males and females before puberty (Leong, 2006), which may contribute to male advantages in jumping and running mechanics and efficiency similar to what has been observed in adults (Daniels & Daniels, 1992; Ferber et al., 2003). Although there are not prepubertal differences in circulating testosterone or hemoglobin concentrations between males and females (McManus & Armstrong, 2011; Senefeld et al., 2020), there are sex‐based differences in lean body mass, cardiac size and function, lung size and function, and pelvic dimensions, all of which may contribute to the greater distances in shot put, javelin throw, and long jump demonstrated by males ages 10‐and‐under compared to females of the same age.

Prepubertal males have been shown to throw farther than females of the same age and to naturally have better overhand throwing technique (Gromeier et al., 2017; Liu & Zhao, 2022; Lombardo & Deaner, 2018; Thomas & French, 1985). Javelin throw is closer to a typical overhand throw than is shot put (Liu et al., 2010), which may help explain why the sex‐based differences in the javelin throw performance are larger than the sex‐based differences in the shot put distance. Javelin throw and long jump also include a running approach aspect with greater running speed contributing to enhanced performance (Hay, 1986; Liu et al., 2010). It has recently been observed that prepubertal males run faster in track sprinting events that females (Atkinson et al., 2024; Brown et al., 2024), which is in agreement with data from fitness testing showing that prepubertal males perform better on sprinting and shuttle run fitness tests (Catley & Tomkinson, 2013; Tomkinson et al., 2017, 2018). Therefore, it is reasonable to conclude that prepubertal males perform better than prepubertal females in shot put, javelin throw, and long jump due to a combination of greater muscle mass, greater muscle strength, greater running speed, and in throwing events greater overhand throwing technique. However, the present data do not preclude the possibility that social and cultural factors can also contribute to the sex‐based differences in shot put, javelin throw, and long jump performance in children age 10‐and‐under.

The differences in athletic performance between male and female children under age 10 are expectedly smaller than the differences observed in adults, primarily due to the testosterone driven changes that occur during male puberty and the estrogen driven changes that occur during female puberty (Bassett et al., 2020; Handelsman, 2017; Handelsman et al., 2018). However, the present and previous data indicate that there are sex‐based differences in shot put, javelin throw, and long jump performance before the onset of puberty, with males consistently throwing and jumping farther than females. It has been suggested that the prepubertal sex‐based differences in physical fitness and athletic performance are due to males participating in a greater amount of physical activity, higher intensity physical activity, and more sports than do females (Hunter et al., 2023). Supporting this suggestion, Chen et al. (Chen et al., 2018) observed in fifth‐grade children that males spend more time engaged in unstructured physical activity than females, but there was not a sex‐based difference in time spent in organized physical education classes, sports, and dance. Hyde et al. (2020) reported that 60.9% of male children and 54.4% of female children engaged in organized sports. Belcher et al. (2010) reported that male children 6–11 years old participate in more moderate to vigorous physical activity than do females. Collectively, these sources support the notion that prepubertal sex‐based differences in athletic performance can at least be partially explained by higher levels of sport participation and more strenuous physical activity in males than in females. However, in 6–7‐year‐old children (Eiberg et al., 2005) and in 8–11‐year‐old children (Dencker et al., 2007), the sex‐based differences in maximal oxygen consumption and body composition were not entirely explained by differences in physical activity. While increased engagement in physical activity and sports can likely improve sports performance (as long as overtraining syndrome is avoided), and some data suggest that male children participate in more sports and physical activity than females, prepubertal sex‐based differences in anatomy and physiology must also be considered as contributing factors to differences in athletic performance between the sexes.

Limitations to the present study are that data are based on track meet records, which offer no insight into the descriptive characteristics of the athletes other than the sex and age group. As it has been previously stated, these data do provide insight into the underlying causes of sex‐based differences in shot put, javelin throw, and long jump performance other than the age group and sex of the athletes. The event records also must be taken at face value, with no knowledge of the accuracy and precision of the measurement used for each event. However, the track meets all occurred under the auspices of USA Track and Field, which has standards for measurements and for athlete eligibility. Although these track meets were labeled as national championships, it is not clear that the best juvenile athletes from across the country are represented among the participants (which is a common challenge in children’s sports due to travel costs and family circumstances associated with attending an event outside the immediate local area). Even though the event records indicate the age group of the children, the ages of the children are not shown in sufficient details to discern biological or psychological maturity status or other factors that can influence sports performance (Cumming et al., 2011). However, these limitations can also be applied to data evaluated by Handelsman (2017), Senefeld et al. (2019), Atkinson et al. (2024), and Brown et al. (2024) and are inherent to the USA Track and Field Youth records (USATF, 2018; USATF, 2019), the AAU Track and Field youth records (AAU), and the School Sport Australia records (School Sport Australia, 2016) and indeed to any comparison of youth sports records.

## 5 CONCLUSION

In conclusion, although some have stated that the prepubertal sex‐based differences in athletic performance are immaterial or absent, the present data indicate that in the 8‐and‐under group and 9–10‐year‐old age group, males achieved greater distances in shot put, javelin throw, and long jump. Although some females in these age groups threw or jumped farther than some males, the average distances for shot put, javelin throw, and long jump for males were farther than for females; the best performing males threw and jumped farther than the best performing females and the average differences between the sexes were larger than within sex differences for the top 4 finalists (i.e., those in closest competition for a medal) for all events and ages except shot put in the 9–10‐year old age group. As throwing and jumping are key components of many sports, these sex‐based differences in shot put, javelin throw, and long jump between males and females ages 10‐and‐under should be considered when sport governing bodies and policy makers consider the issue of sex‐segregated sporting categories.

## CONFLICT OF INTEREST STATEMENT

Dr. Brandon Shaw and Dr. Ina Shaw declare that they have no conflicts of interest. Dr. Greg Brown declares that he is currently serving as an expert witness in seven different legal cases in the United States regarding the inclusion of transgender identified males (i.e., trans women) in female sports. No funding that supported this project as part of service as an expert witness was received and his service as an expert witness is not reliant upon publishing this manuscript.

## Data Availability

These data were derived from resources available in the public domain at https://www.athletic.net/.
